# MITOCHONDRIAL COMMUNICATION AND AGING

**DOI:** 10.1093/geroni/igac059.1141

**Published:** 2022-12-20

**Authors:** Changhan Lee

## Abstract

Mitochondria evidently originate from endosymbiotic bacteria that presumably provided several advantages for eukaryotic life. For the past 1–2 billion years, mitochondria co-evolved with the ancestral cell to coordinate various cellular functions. Coordination requires communication and mitochondrial signaling has been shown to be vital to cellular fitness and aging. In this symposium, the speakers will discuss the role of mitochondria as a signaling organelle and their impact on key cellular functions in the context of aging. Consistent with emerging evidence of the complexity and sophistication of mitochondrial communication mechanisms, some of the mitochondrial-to-nuclear communication modes, including nuclear transcriptional programs and cellular signaling networks, that regulate molecular and cellular processes to promote fitness will be discussed. Dr. Chen will discuss how a mitochondrial metabolic checkpoint can regulate stem cell quiescence and maintenance that is important to stem cell aging. Dr. Picard will discuss the impact of mitochondrial stress on increased energetic cost of living (i.e. hypermetabolism) and cellular lifespan. Finally, Dr. Lee will discuss how signaling peptides that are encoded withing the mitochondrial genome regulate cellular homeostasis, increase physiological resilience, and promote healthy aging.

